# Effects of a Short-Term Interactive Biofeedback Therapy Using the Lumbar-Type Hybrid Assistive Limb in Older Adults With Reduced Physical Function

**DOI:** 10.7759/cureus.104505

**Published:** 2026-03-01

**Authors:** Mitsutaka Yakabe, Yoshihiro Yasunaga, Yoshihiro Zenita, Shoya Matsumoto, Tatsuya Hosoi, Sumito Ogawa

**Affiliations:** 1 Department of Geriatric Medicine, Graduate School of Medicine, The University of Tokyo, Tokyo, JPN; 2 Executive Office, Japan Clinical Recurrent Education and Research Center, Aichi, JPN

**Keywords:** cybernics, finger-floor distance, frailty, gait speed, lumbar-type hybrid assistive limb, rehabilitation, sit to stand-5, timed up and go

## Abstract

Aim: Efficient rehabilitation strategies for older adults with reduced physical function have not been firmly established. The lumbar-type Hybrid Assistive Limb (HAL) is a wearable cyborg device designed to support trunk function and sit-to-stand movements, and can be conceptualized as an interactive biofeedback therapy platform that may also enhance gait performance. However, few studies have examined the effects of very short-duration exercise protocols using this device in this population. Therefore, we investigated the effects of a brief lumbar-type HAL-based therapy program in older adults with impaired physical function.

Methods: This was a single-arm interventional study. Participants were community-dwelling older adults aged ≥65 years attending day-care rehabilitation at Chigusa Sawayaka Clinic (Nagoya, Aichi, Japan). The therapy program consisted of supervised sessions using the lumbar-type HAL for 10 minutes per session, once per week for four consecutive weeks (total HAL-based therapy time: 40 minutes). The primary outcome was Timed Up and Go (TUG) time. Secondary outcomes were 10-m gait speed, stride length, five-repetition sit-to-stand (SS5) time, and finger-floor distance. All outcomes were assessed at 11 predefined time points: one week before session 1; immediately before and after each of the four sessions; and one week and one month after session 4. These repeated measurements were used to evaluate the effects of the short-duration HAL-based therapy program.

Results: Ten participants (mean age 82.3 ± 10.4 years, range 65-96; five men and five women) completed the program. From the first to the final assessment, TUG time decreased from 18.47 ± 4.33 s to 14.45 ± 4.10 s, and SS5 time decreased from 20.00 ± 4.18 s to 12.58 ± 2.51 s, while gait speed increased from 0.80 ± 0.26 m/s to 0.95 ± 0.35 m/s. In analyses using a linear mixed-effects model, TUG time showed a significant acute improvement from immediately before to immediately after session 1 (Δ -1.91 s, 95% CI -3.57- -0.24), and the mean within-session change across all four sessions was also significant (Δ -1.10 s, 95% CI -1.93- -0.27). These improvements were maintained at one week (Δ -3.49 s, 95% CI -5.15- -1.83) and one month (Δ -3.92 s, 95% CI -5.58- -2.26) after session 4. Gait speed and SS5 times likewise showed significant reductions both after session 1 and in the average within-session effect across the four sessions, and these improvements were preserved at one week and one month after the final session.

Conclusions: Short-duration interactive biofeedback therapy delivered with a lumbar-type HAL was associated with improvements in multiple aspects of physical function in older adults with reduced physical capacity. Future research should evaluate the effects of HAL in randomized controlled trials with a larger sample size and longer follow-up.

## Introduction

The global population is ageing. By the 2050s, the number of older adults is projected to reach 2 billion, accounting for approximately 22% of the world’s population [1]. Therefore, broader measures that support healthy ageing and continued participation in society are becoming increasingly important to mitigate the macroeconomic burden associated with population ageing.

Sarcopenia is a syndrome characterized by a systemic and progressive loss of skeletal muscle mass and strength with advancing age [2]. Frailty is a condition in which physiological reserve across multiple organ systems is diminished, making individuals more likely to develop dependency and to require long-term care in response to minor illnesses or stress [3]. Sarcopenia and frailty are prototypical geriatric syndromes that have been consistently associated with increased risks of disability, hospitalization, and all-cause mortality in older adults, and therefore contribute to reduced healthy life expectancy [4]. Moreover, sarcopenia and frailty share a common biological substrate of impaired muscle mass, strength, and physical performance, frequently coexist and appear to reinforce one another; declines in muscle function promote frailty, while frailty accelerates inactivity and deconditioning, establishing a self-perpetuating vicious cycle of deteriorating physical function [5].

One of the key strategies for improving both sarcopenia and frailty is structured exercise or rehabilitation, particularly multicomponent and resistance-based programs that target muscle strength, balance, and mobility [6]. Frailty has been suggested to be a potentially reversible condition, and early intervention may enable individuals to return to a more robust state [7]. However, rehabilitation in older adults poses several challenges. Many older adults live with multimorbidity, and their comorbid conditions can restrict the type or intensity of exercise that is safe and feasible. In addition, because baseline physical function is often reduced, high-intensity or long-duration rehabilitation programs are often not tolerated. In super-aged societies, there is therefore a growing need for rehabilitation strategies that are brief, low-burden, yet efficient enough to be implemented even in older adults with substantially impaired physical function.

The Hybrid Assistive Limb (HAL) is a wearable cyborg device that detects bioelectrical signals from the skin surface and assists voluntary movements via a cybernics interactive biofeedback loop. Cybernics is an innovative academic field that integrates humans, robots, and information systems [8]. Within this framework, the cybernics interactive biofeedback loop constitutes a therapeutic approach in which the wearer’s voluntary motor intent, detected as bioelectrical signals (e.g., motor unit potentials), is directly linked to robotic assistance provided by HAL [9]. In this loop, assistance is dynamically modulated according to the user’s motor intention, and the resulting movement generates sensory feedback from proprioceptors that is returned to the central nervous system. This bidirectional exchange of neural and mechanical information enables repeated successful voluntary movements without imposing excessive neuromuscular load or fatigue, thereby facilitating efficient motor relearning and functional improvement. Consistent with this concept, a recent systematic review demonstrated that actively controlled exoskeletons that detect bioelectrical signals, such as HAL, are associated with superior functional recovery and evidence of activity-dependent neuroplasticity compared with passively controlled devices [10]. In 2013, a small pilot study of the HAL in patients with stroke demonstrated the feasibility of HAL-based therapy [11]. In a five-week randomized controlled trial of community-dwelling older adults with mobility limitations, use of the lumbar-type HAL led to significant improvements in several functional domains compared with controls [12]. In a pilot study involving participants with locomotive syndrome, a 12-session program using the lumbar-type HAL-comprising sit-to-stand practice, trunk flexion-extension, and gait training-produced significant improvements in mobility outcomes, with no adverse events reported [13]. Furthermore, HAL has been reported to improve functional outcomes during postoperative rehabilitation following hip fracture surgery [14].

Thus, over the past decade, HAL has been suggested as a potentially useful tool in rehabilitation; however, there are few studies evaluating very short, low-burden training protocols that are tolerable even for physically frail older adults. Therefore, we conducted an exploratory, single-arm interventional study in older adults attending outpatient day-care rehabilitation to evaluate the short-term feasibility and preliminary effects of a lumbar-type HAL-based interactive biofeedback therapy.

## Materials and methods

Study design and setting

This single-arm interventional study included patients enrolled between May and July 2025 who underwent therapy using a lumbar-type HAL at Chigusa Sawayaka Clinic, an outpatient day-care rehabilitation facility in Nagoya, Aichi, Japan. The final follow-up assessments (up to one month after the fourth session) were conducted in August 2025.

Participants and eligibility

Eligible participants were patients aged ≥65 years who attended day-care rehabilitation at the clinic during the study period and had a Timed Up and Go (TUG) time of ≥13.5 s and <30 s. Eligibility was not restricted by sex, underlying diagnoses, or medications. Participants were required to be able to safely complete all outcome assessments described below; the use of customary walking aids (e.g., a cane or rollator) was permitted.

Patients were excluded if they (ⅰ) could not safely perform one or more assessments, (ii) had severe cognitive impairment or communication difficulties that prevented understanding of device use or adherence to therapist instructions, (iii) had an acute medical condition or other contraindication judged by the treating team to make exercise unsafe, (iv) did not complete the scheduled therapy program, or (v) did not provide written informed consent.

Before starting the therapy, all patients received a detailed explanation of the lumbar-type HAL, and only those who agreed to undergo HAL-based therapy were included in the study. Written informed consent was obtained from all participants.

HAL-based therapy and assessment schedule

At Chigusa Sawayaka Clinic, a structured exercise program using a lumbar-type HAL was provided as part of an outpatient day-care rehabilitation service under the Japanese long-term care insurance system.

The duration was set to within 10 minutes, including the time from donning to doffing the HAL device, and the actual HAL-based therapy session lasted approximately seven minutes. Before each session, the therapist confirmed proper device fitting (belt position, alignment of the hip joint axis, fastening, and range-of-motion limits) according to the manufacturer’s instructions (CYBERDYNE Inc.,Tsukuba, Japan; lumbar-type HAL). Surface electrodes and sensors were placed as specified by the manufacturer to detect bioelectrical signals related to hip flexion/extension. A brief calibration was then performed with the participant in standing and seated positions to (i) verify stable signal detection, (ii) confirm correct directionality of assist for flexion/extension, and (iii) ensure that the device responded appropriately during a few practice repetitions of sit-to-stand and trunk movements. If signal detection was unstable, electrode placement and skin preparation were adjusted, and calibration was repeated. HAL assistance was provided in a voluntary-control mode (i.e., assist was triggered in response to the participant’s detected bioelectrical signals). For each session, an assist level was selected to allow for smooth and safe completion of movements without requiring physical lifting by the therapist. Each session was supervised by a licensed physical therapist trained in HAL operation. The exercise set consisted of repeated sit-to-stand, trunk flexion/extension, and supported squats performed at a self-selected pace with short rests as required.

After consent was obtained, each participant underwent HAL-based therapy once per week for four consecutive weeks (four sessions in total).

**Figure 1:**
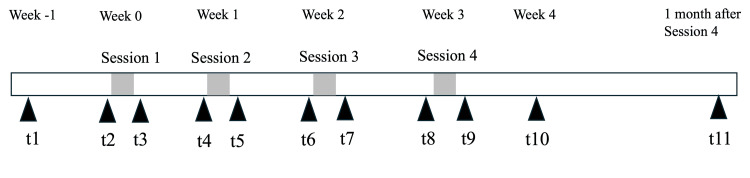
Study timeline and assessment schedule. Time points: t1 = one week before session 1; t2 and t3 = immediately before and after session 1; t4 and t5 = immediately before and after session 2; t6 and t7 = immediately before and after session 3; t8 and t9 = immediately before and after session 4; t10 = one week after session 4; t11 = one month after session 4.

Clinical outcomes were assessed repeatedly across the study period. The first assessment was performed one week before the first HAL session. Thereafter, all outcomes were measured immediately before and after each of the four weekly sessions, as well as one week and one month after the fourth session, resulting in 11 assessment time points in total (Figure 1). These time points were defined as t1 (one week before session 1); t2 and t3 (immediately before and after session 1); t4 and t5 (immediately before and after session 2); t6 and t7 (immediately before and after session 3); t8 and t9 (immediately before and after session 4); t10 (one week after session 4); and t11 (one month after session 4). All outcome assessments were performed by the treating therapists; therefore, assessors were not blinded. Each outcome was assessed once. Because multiple outcomes were assessed, participants were given sufficient rest between measurements to minimize fatigue.

Participants continued their usual rehabilitation program while receiving HAL-based therapy and did not change their lifestyle habits during the study period.

Primary outcome

The primary outcome was TUG time. TUG time (s) was measured from the verbal “go” cue to the moment the participant sat back and made contact with the chair after standing, walking 3 m, turning, and returning to sit [15]. TUG time was assessed at the same 11 time points (t1-t11).

Secondary outcomes

Gait Speed

Gait speed (m/s) was measured on a straight, level walkway using a 10-meter walk test protocol with acceleration/deceleration zones [16]. A 16-m course was marked with a 3-m acceleration zone, a 10-m timed section, and a 3-m deceleration zone. Participants were instructed to “walk as fast as you can safely walk, without running.” Timing was obtained once over the 10-m section with a handheld stopwatch, and speed (m/s) was calculated as distance/time. Assistive devices customarily used for ambulation were permitted to ensure consistency across tests. Gait speed was also assessed at all 11 time points (t1-t11) described above.

Stride Length

Stride length was assessed concurrently with gait speed. During each gait speed trial, we counted the number of steps required to walk the 10-m section and calculated stride length (m) by dividing 10 m by the corresponding step count, such that larger values indicate a longer stride. Stride length was also assessed at the same 11 time points (t1-t11).

Sit to Stand-5 (SS5)

Participants sat on a standard armless chair of fixed height, with their feet placed flat on the floor and their arms folded across their chests. On the verbal cue “go,” they were instructed to stand up fully and sit down five times as quickly as possible without using their arms for support. The time (s) required to complete five repetitions were recorded with a stopwatch, with shorter times indicating better performance [17]. SS5 time was also assessed at the same 11 time points (t1-t11).

Finger-Floor Distance (FFD)

Participants stood barefoot on a flat surface with their feet shoulder-width apart and their knees fully extended. They were then instructed to slowly bend forward and reach toward the floor with both hands, without bouncing or flexing the knees. The vertical distance between the tip of the middle finger and the floor was measured using a tape measure; values with the fingertips above the floor were recorded as positive distances, and those with the fingertips reaching beyond the floor level were recorded as negative distances [18]. FFD was also assessed at the same 11 time points (t1-t11).

Statistical analysis

Repeated measures were analyzed with a linear mixed-effects model (LMM) with time (11 levels, t1-t11) as a categorical fixed effect and a participant-specific random intercept. No random slopes were included (random-intercepts-only model). The primary LMM assumed independent residuals conditional on the random intercept. Models were fitted by restricted maximum likelihood, and denominator degrees of freedom were estimated using the Kenward-Roger method. Estimated marginal means (EMMs) were obtained for each time point, and the following pre-specified contrasts were tested: (i) acute within-session effects (post minus pre for each session) and their average across the four sessions; (ii) a cumulative between-session trend across the four pre-session (t2, t4, t6, t8) means, using linear contrast weights −3, −1, +1, +3; (iii) maintenance after the final session (immediately after session 4 vs. one-week and one-month follow-up assessments); and (iv) overall change from the first pre-session assessment (t2) to immediately after session 4 (t9), one week (t10), and one month (t11) after session 4.

Two-sided tests were performed with α = 0.05. The family-wise error rate across this primary set of contrasts was controlled using Holm’s step-down procedure, and 95% confidence intervals (CIs) were reported with Bonferroni adjustment for simultaneous inference. Holm-adjusted p-values and Bonferroni-adjusted simultaneous 95% CIs were applied within each outcome across the 11 pre-specified contrasts; no additional adjustment was applied across multiple outcomes. Descriptive plots showing mean ± standard deviation (SD) at each time point and individual trajectories were produced. Sensitivity analyses examined the robustness of the results to the assumed correlation structure and modeling assumptions, including an autoregressive AR(1) residual correlation model and cluster-robust standard errors (CR2) with participants treated as clusters. For the AR(1) sensitivity analyses, models were refitted using nlme::lme with corAR1(form = ~ time | ID) and heteroscedastic residual variances by time point using weights = varIdent(form = ~ 1 | time). For the CR2 sensitivity analyses, cluster-robust variance-covariance matrices were computed using clubSandwich::vcovCR (type = "CR2") with participant as the clustering unit, and EMMs/contrasts were re-estimated using the robust variance-covariance matrix; inference was based on asymptotic (z) tests.

Secondary outcomes (gait speed, stride length, SS5, and FFD) were analyzed exploratorily using the same LMM framework as for the primary outcome, with time as a categorical fixed effect and a participant-specific random intercept. The same set of pre-specified contrasts was applied to each secondary outcome. Given the exploratory nature of these analyses, p-values and confidence intervals were interpreted descriptively without additional adjustment for multiple comparisons. Models were re-estimated under an AR(1) residual correlation structure and with CR2 to evaluate robustness to the assumed covariance structure and potential model misspecification.

P-values < 0.05 were considered significant. All statistical analyses were performed using R 4.3.3 software (R Foundation for Statistical Computing, Vienna, Austria). The primary LMMs were fitted using lme4/lmerTest, EMMs and contrasts were obtained using emmeans, AR(1) models were fitted using nlme, and CR2 robust standard errors were computed using clubSandwich.

## Results

Thirteen patients were screened for eligibility. Eleven patients who met the inclusion criteria of a TUG time of ≥13.5 s and <30 s were recruited, provided written informed consent, and completed the baseline assessment (t1). One participant sustained a fracture before the pre-session assessment for session 1 (t2) and was withdrawn. The remaining 10 participants completed the HAL-based therapy program from start to finish, and no further dropouts occurred between the first therapy session and the final follow-up assessment (t11). Their baseline characteristics are shown in Table 1.

**Table 1:** Characteristics of participants (n = 10, 100%).

No.	Age	Sex	Care-need level	Primary diagnoses
1	71	Female	Care Level 4	Sequelae of a left putaminal hemorrhage (right hemiparesis)
2	95	Female	Care Level 1	Multiple lumbar vertebral compression fractures
3	79	Female	Support Level 2	History of right femoral neck fracture; bilateral knee osteoarthritis; breast cancer; hypertension; diabetes mellitus; cataracts.
4	75	Male	Care Level 1	Post-polio syndrome; disuse syndrome
5	89	Female	Support Level 1	History of right humeral fracture; knee osteoarthritis; subarachnoid hemorrhage; myocardial infarction.
6	65	Female	Care Level 2	Sequelae of hemorrhagic infarction (right hemiparesis).
7	91	Male	Care Level 1	Right medial medullary infarction; cervical spondylosis; dyslipidemia; hypertension; chronic pancreatitis; angina pectoris.
8	82	Male	Support Level 1	Left lacunar infarction; right incomplete hemiparesis; diabetes mellitus; hypertension; benign prostatic hyperplasia.
9	80	Male	Support Level 2	Right middle cerebral artery territory cerebral infarction; hypertension; dyslipidemia.
10	96	Male	Support Level 1	Disuse syndrome; type 2 diabetes mellitus; dyslipidemia; chronic kidney disease

**Figure 2:**
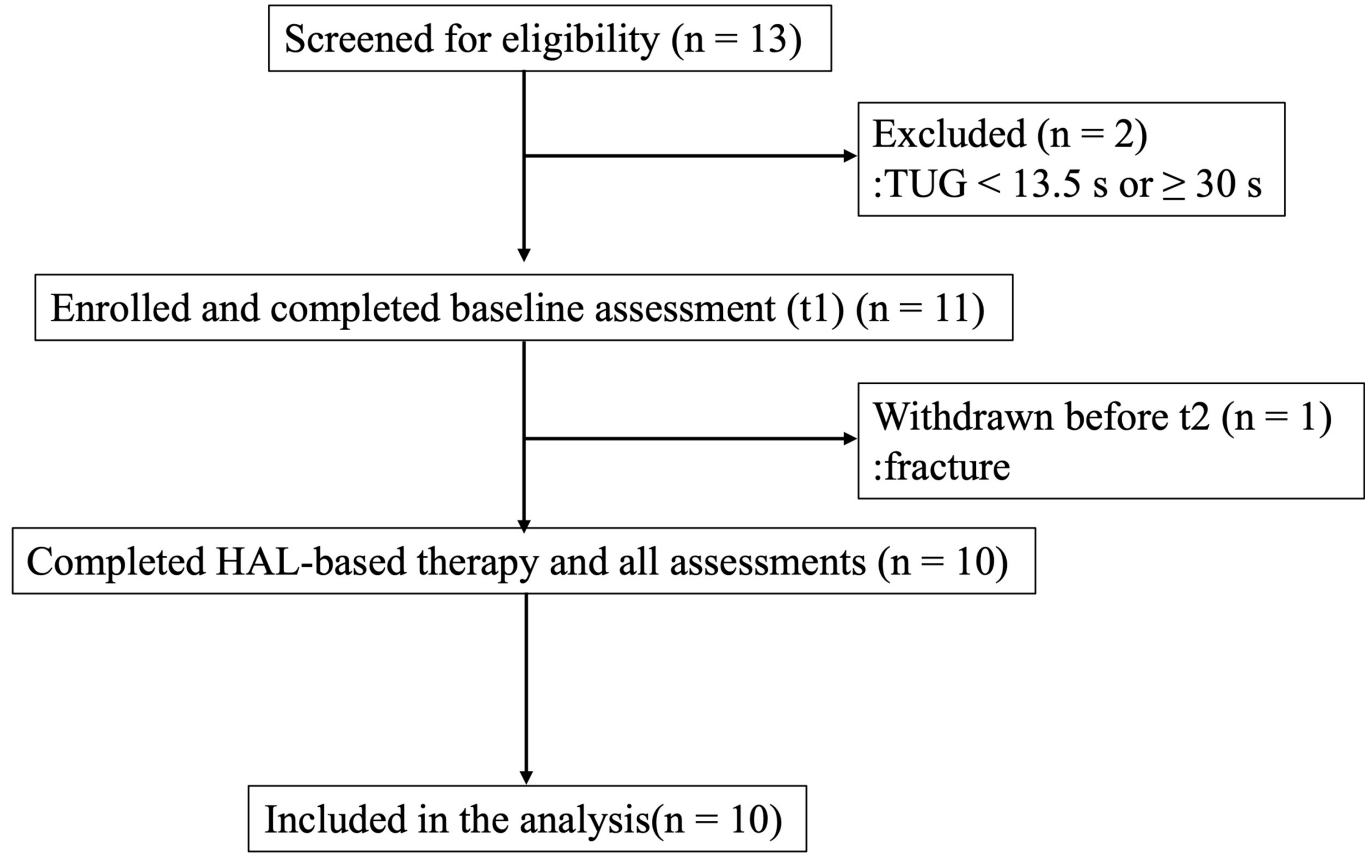
Participant flow diagram. TUG = Timed Up and Go; HAL = Hybrid Assistive Limb.

The mean age was 82.3 ± 10.4 years (range, 65-96), and the group consisted of five men and five women. Nine participants (90%) were classified as requiring support or care level 1-2 under the Japanese long-term care insurance system. A participant flow diagram is shown in Figure 2.

Unadjusted descriptive statistics for all outcomes at each time point (t1-t11) are presented in Table 2. When comparing t1 and t11, TUG time decreased from 18.47 ± 4.33 s to 14.45 ± 4.10 s, and SS5 time decreased from 20.00 ± 4.18 s to 12.58 ± 2.51 s, while gait speed increased from 0.80 ± 0.26 m/s (t1) to 0.95 ± 0.35 m/s (t11).

**Table 2:** Unadjusted descriptive statistics for all outcomes at each of the 11 time points. This table shows the unadjusted descriptive statistics - arithmetic mean, standard deviation, minimum, and maximum - for all outcomes at each of the 11 time points (t1–t11). Time points: t1 = one week before session 1; t2 = immediately before session 1; t3 = immediately after session 1; t4 = immediately before session 2; t5 = immediately after session 2; t6 = immediately before session 3; t7 = immediately after session 3; t8 = immediately before session 4; t9 = immediately after session 4; t10 = one week after session 4; t11 = one month after session 4. TUG = Timed Up and Go; SS5 = five-repetition sit-to-stand; FFD = finger-floor distance.

Time	TUG	Gait speed	Stride length	SS5	FFD
t1	18.47 ± 4.33 (13.50–25.07)	0.80 ± 0.26 (0.40–1.07)	0.43 ± 0.11 (0.27–0.56)	20.00 ± 4.18 (14.91–28.06)	-13.1 ± 12.2 (-32–10)
t2	18.37 ± 4.52 (12.91–27.09)	0.79 ± 0.28 (0.32–1.16)	0.41 ± 0.10 (0.25–0.56)	17.80 ± 4.22 (11.06–24.94)	-13.7 ± 12.2 (-31–10)
t3	16.46 ± 4.94 (11.88–25.72)	0.88 ± 0.30 (0.43–1.27)	0.43 ± 0.10 (0.27–0.56)	15.16 ± 3.53 (9.41–21.22)	-9.0 ± 11.2 (-26–13)
t4	17.47 ± 5.47 (11.85–28.28)	0.84 ± 0.31 (0.33–1.26)	0.42 ± 0.10 (0.24–0.56)	16.13 ± 3.98 (10.66–23.34)	-10.9 ± 10.9 (-28–9)
t5	16.52 ± 5.02 (11.00–26.60)	0.89 ± 0.30 (0.42–1.27)	0.43 ± 0.09 (0.28–0.56)	14.00 ± 2.57 (9.10–18.43)	-6.5 ± 11.2 (-24–14)
t6	16.91 ± 4.93 (11.94–25.34)	0.87 ± 0.31 (0.43–1.26)	0.43 ± 0.10 (0.26–0.56)	14.88 ± 3.49 (9.47–22.57)	-9.7 ± 14.1 (-27–12)
t7	16.30 ± 5.25 (11.66–25.62)	0.94 ± 0.33 (0.44–1.33)	0.43 ± 0.10 (0.28–0.56)	13.85 ± 3.96 (7.47–21.35)	-6.9 ± 13.2 (-23–15)
t8	16.16 ± 5.02 (11.34–26.71)	0.92 ± 0.33 (0.42–1.27)	0.42 ±0.11 (0.26–0.56)	14.36 ± 2.76 (10.15–18.72)	-9.4 ± 13.5 (-27–13)
t9	15.24 ± 4.35 (10.85–23.06)	0.95 ± 0.32 (0.45–1.31)	0.43 ± 0.09 (0.29–0.56)	13.23 ± 2.54 (9.75–17.84)	-7.0 ± 14.6 (-27–15)
t10	14.88 ± 4.33 (10.43–23.03)	0.96 ± 0.33 (0.43–1.33)	0.44 ± 0.11 (0.26–0.59)	11.96 ± 2.55 (7.41–15.37)	-9.8 ± 14.5 (-30–11)
t11	14.45 ± 4.10 (10.28–22.31)	0.95 ± 0.35 (0.42–1.37)	0.44 ± 0.10 (0.26–0.56)	12.58 ± 2.51 (10.03–16.15)	-10.6 ± 12.2 (-27–9)

To assess whether these observed changes over time were statistically significant, we applied LMMs. These longitudinal changes are summarized in Table 3, which presents the EMMs (with 95% CIs) of all outcomes at each of the 11 time points.

**Table 3:** EMMs for all outcomes at each time point EMMs with 95% CIs from an LMM with time (11 levels) as a fixed effect and participant-specific random intercepts are presented for all outcomes. Time points: t1 = one week before session 1; t2 = immediately before session 1; t3 = immediately after session 1; t4 = immediately before session 2; t5 = immediately after session 2; t6 = immediately before session 3; t7 = immediately after session 3; t8 = immediately before session 4; t9 = immediately after session 4; t10 = one week after session 4; t11 = one month after session 4. TUG = Timed Up and Go; SS5 = five-repetition sit-to-stand; FFD = finger-floor distance; EMM = estimated marginal mean; LMM: linear mixed-effects model.

Time	TUG	Gait speed	Stride length	SS5	FFD
t1	18.47 (15.52–21.43)	0.803 (0.610–0.996)	0.427 (0.364–0.490)	20.00 (17.91–22.08)	-13.1 (-21.0– -5.2)
t2	18.37 (15.41–21.32)	0.786 (0.593–0.979)	0.410 (0.347–0.473)	17.80 (15.71–19.89)	-13.7 (-21.6– -5.8)
t3	16.46 (13.50–19.42)	0.879 (0.686–1.072)	0.426 (0.363–0.489)	15.16 (13.08–17.25)	-9.0 (-16.9– -1.1)
t4	17.47 (14.51–20.43)	0.839 (0.646–1.032)	0.420 (0.357–0.483)	16.13 (14.04–18.22)	-10.9 (-18.8– -3.0)
t5	16.52 (13.57–19.48)	0.888 (0.695–1.081)	0.428 (0.365–0.491)	14.00 (11.91–16.09)	-6.5 (-14.4–1.4)
t6	16.91 (13.96–19.87)	0.873 (0.680–1.066)	0.426 (0.363–0.489)	14.88 (12.79–16.97)	-9.7 (-17.6– -1.8)
t7	16.30 (13.35–19.26)	0.941 (0.748–1.134)	0.430 (0.367–0.493)	13.85 (11.76–15.94)	-6.9 (-14.8–1.0)
t8	16.16 (13.21–19.12)	0.916 (0.723–1.109)	0.423 (0.360–0.486)	14.36 (12.27–16.45)	-9.4 (-17.3– -1.5)
t9	15.24 (12.28–18.19)	0.952 (0.759–1.145)	0.426 (0.363–0.489)	13.23 (11.14–15.32)	-7.0 (-14.9–0.9)
t10	14.88 (11.92–17.83)	0.956 (0.763–1.149)	0.441 (0.378–0.504)	11.96 (9.87–14.04)	-9.8 (-17.7– -1.9)
t11	14.45 (11.49–17.40)	0.950 (0.757–1.143)	0.435 (0.372–0.498)	12.58 (10.49–14.66)	-10.6 (-18.5– -2.7)

**Figure 3:**
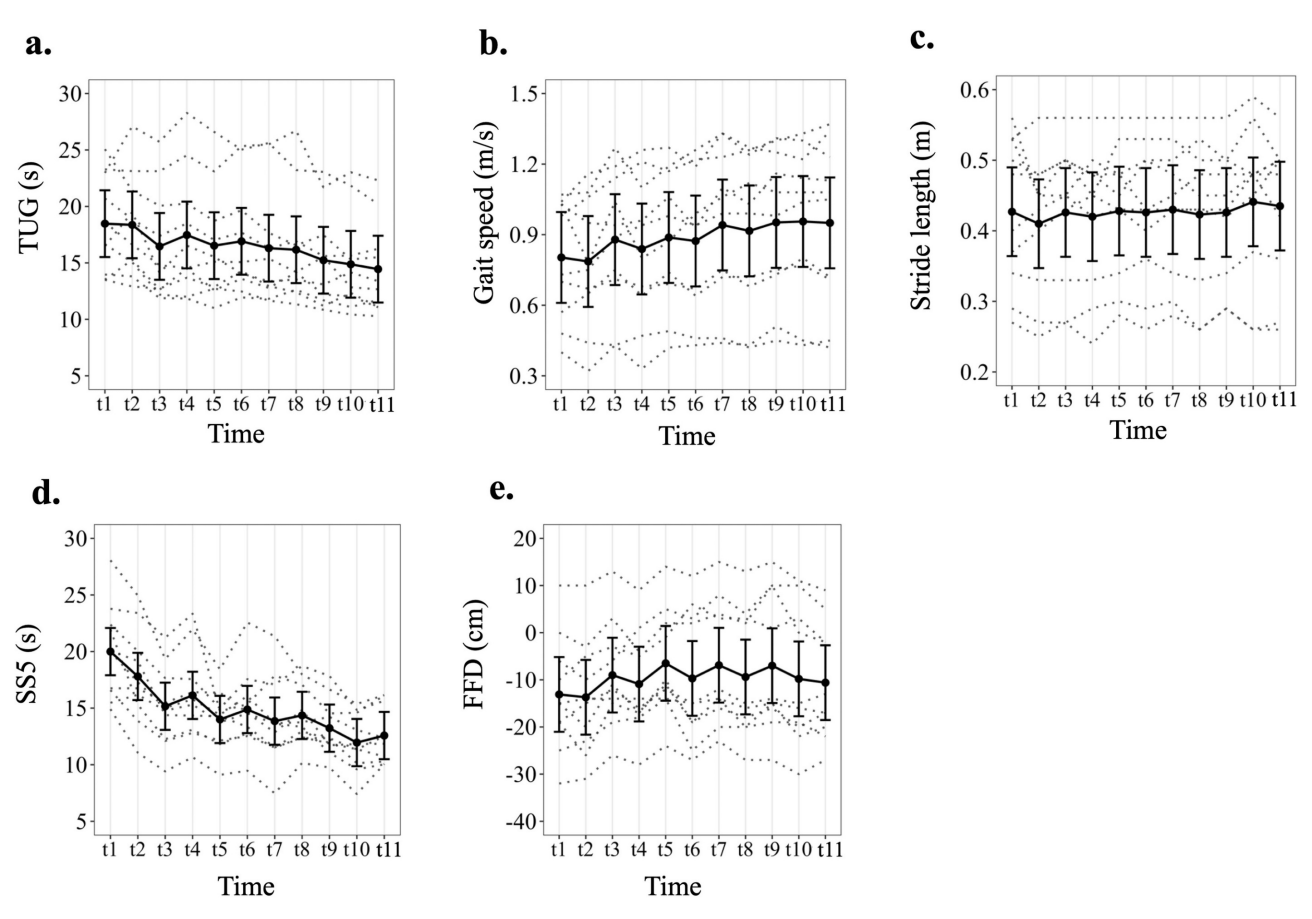
Longitudinal profiles of all outcomes; EMMs with 95% CIs. Panels: (a) Timed Up and Go; (b) gait speed; (c) stride length; (d) five-repetition sit-to-stand; (e) finger-floor distance. Black circles and solid lines show EMMs from a linear mixed-effects model with time (11 levels) as a fixed effect and a participant-specific random intercept. Vertical bars are 95% CIs; dotted gray lines show individual trajectories (n = 10, 100%). Time points: t1 = one week before session 1; t2 = immediately before session 1; t3 = immediately after session 1; t4 = immediately before session 2; t5 = immediately after session 2; t6 = immediately before session 3; t7 = immediately after session 3; t8 = immediately before session 4; t9 = immediately after session 4; t10 = one week after session 4; t11 = one month after session 4. EMM = estimated marginal mean.

Figure 3 illustrates the longitudinal profiles of all outcomes, displaying the EMMs (with 95% CIs) at each time point.

For the primary outcome of TUG time, comparison of values immediately before and after session 1 showed a statistically significant improvement after Holm adjustment (t3−t2 = -1.91 s, 95% CI -3.57- -0.24, P = 0.007; Table 4). No statistically significant within-session changes were observed for sessions 2-4. However, the average within-session change across all four sessions was significant (average immediate effect = -1.10 s, 95% CI -1.93- -0.27, P = 0.001). The linear trend across the four pre-session baseline values was -7.16 s (95% CI -12.42- -1.91, P < 0.001), indicating a significant downward trend in TUG time over the course of the program. Pre-session values exhibited a significant downward linear trend, consistent with cumulative gains over time. Improvements observed immediately after session 4 were maintained at one week and one month (t10−t9 = -0.36 s and t11−t9 = -0.79 s; neither was significant). Compared with t2, TUG time remained lower at t9, t10, and t11 by -3.13, -3.49, and -3.92 s (all P < 0.001). Sensitivity analysis for the TUG time using an AR(1) residual correlation structure and CR2 robust standard errors yielded effect estimates that were directionally consistent with the primary linear mixed-effects models. As the sample size was small (n = 10), statistical inference was interpreted cautiously; notably, some contrasts showed borderline sensitivity to modeling assumptions (e.g., the pre-intervention linear trend for TUG was significant under AR(1) but not under CR2). In contrast, the main within-participant improvements from baseline to post-intervention and follow-up remained statistically significant across model specifications for TUG.

**Table 4:** Prespecified contrasts for each outcome. P values are Holm-adjusted, and CIs are Bonferroni-adjusted simultaneous 95% CIs across 11 contrasts. *P<0.05 was considered significant. The row “Linear trend across pre-session baselines” tests whether baseline performance increases in a linear fashion across sessions by applying contrast weights −3, −1, +1, +3 to the EMMs at t2, t4, t6, t8, respectively. Time points: t1 = one week before session 1; t2 = immediately before session 1; t3 = immediately after session 1; t4 = immediately before session 2; t5 = immediately after session 2; t6 = immediately before session 3; t7 = immediately after session 3; t8 = immediately before session 4; t9 = immediately after session 4; t10 = one week after session 4; t11 = one month after session 4. TUG = Timed Up and Go; SS5 = five-repetition sit-to-stand; FFD = finger-floor distance; EMM = estimated marginal mean.

Contrast	TUG		Gait speed		Stride length		SS5		FFD	
	Estimate (95%CI)	P-value	Estimate (95%CI)	P-value	Estimate (95%CI)	P-value	Estimate (95%CI)	P-value	Estimate (95%CI)	P-value
Immediate effect after session 1 (t3-t2)	-1.91 (-3.57– -0.24)	0.007*	0.093 (0.014–0.172)	0.005*	0.016 (-0.013–0.045)	1.000	-2.64 (-4.50– -0.77)	<0.001*	4.70 (0.82–8.58)	0.005*
Immediate effect after session 2 (t5-t4)	-0.944 (-2.61–0.72)	0.535	0.049 (-0.030–0.128)	0.308	0.008 (-0.021–0.037)	1.000	-2.13 (-3.99– -0.27)	0.006*	4.40 (0.53–8.28)	0.009*
Immediate effect after session 3 (t7-t6)	-0.61 (-2.27–1.05)	0.594	0.068 (-0.011–0.147)	0.071	0.004 (-0.025–0.033)	1.000	-1.03 (-2.89–0.84)	0.256	2.80 (-1.08–6.68)	0.121
Immediate effect after session 4 (t9-t8)	-0.93 (-2.59–0.73)	0.535	0.036 (-0.043–0.115)	0.582	0.003 (-0.026–0.032)	1.000	-1.13 (-2.99–0.73)	0.256	2.40 (-1.48–6.28)	0.121
Average immediate effect across sessions 1–4	-1.10 (-1.93– -0.27)	0.001*	0.062 (0.022–0.101)	<0.001*	0.008 (-0.007–0.022)	1.000	-1.73 (-2.66– -0.80)	<0.001*	3.58 (1.64–5.51)	<0.001*
Lenear trend across pre-session baselines	-7.16 (-12.42– -1.91)	<0.001*	0.424 (0.175–0.673)	<0.001*	0.045 (-0.047–0.137)	1.000	-11.58 (-17.47– -5.68)	<0.001*	14.10 (1.84–26.36)	0.009*
Change from post-session 4 to 1 week (t10 − t9)	-0.36 (-2.02–1.30)	0.594	0.004 (-0.075–0.083)	1.000	0.015 (-0.014–0.044)	1.000	-1.27 (-3.14–0.59)	0.212	-2.80 (-6.68–1.08)	0.121
Change from post-session 4 to 1 month (t11 − t9)	-0.79 (-2.45–0.87)	0.535	-0.002 (-0.081–0.077)	1.000	0.009 (-0.020–0.038)	1.000	-0.65 (-2.52–1.21)	0.322	-3.60 (-7.48–0.28)	0.042*
Change from first pre-session to post-session 4 (t9 − t2)	-3.13 (-4.79– -1.47)	<0.001*	0.166 (0.087–0.245)	<0.001*	0.016 (-0.013–0.045)	1.000	-4.57 (-6.44– -2.71)	<0.001*	6.70 (2.82–10.58)	<0.001*
Change from first pre-session to 1 week (t10 − t2)	-3.49 (-5.15– -1.83)	<0.001*	0.170 (0.091–0.249)	<0.001*	0.031 (0.002–0.060)	0.027*	-5.84 (-7.71– -3.98)	<0.001*	3.90 (0.02–7.78)	0.026*
Change from first pre-session to 1 month (t11 − t2)	-3.92 (-5.58– -2.26)	<0.001*	0.164 (0.085–0.243)	<0.001*	0.025 (-0.004–0.054)	0.146	-5.22 (-7.09– -3.36)	<0.001*	3.10 (-0.78–6.98)	0.093

The results of LMMs for secondary outcomes are also summarized in Table 4. For gait speed, we observed clear improvements over time: there were significant acute within-session gains after session 1 (t3−t2 = 0.093, 95% CI 0.014-0.172, P = 0.005), as well as significant average immediate effects across sessions 1-4 and strong linear trends across the four pre-session baselines (average immediate effect = 0.062, 95% CI 0.022-0.101). In addition, changes from the first pre-session assessment to immediately after session 4, and to the one-week and one-month follow-up assessments, were all statistically significant (t9−t2 = 0.166, t10−t2 = 0.170, and t11−t2 = 0.164; all P < 0.001). For SS5, we also observed clear improvements over time: there were significant acute within-session gains after session 1 (t3−t2 = -2.64, 95% CI -4.50- -0.77, P = 0.005), as well as significant average immediate effects across sessions 1-4 and strong linear trends across the four pre-session baselines (average immediate effect = -1.73, 95% CI -2.66- -0.80, P < 0.001). Changes from the first pre-session assessment to immediately after session 4, and to the one-week and one-month follow-up assessments, were all statistically significant (t9−t2 = -4.57, t10−t2 = -5.84, and t11−t2 = -5.22; all P < 0.001). In contrast, stride length showed only modest changes; none of the within-session contrasts reached significance, and among the longer-term contrasts, only the change from the first pre-session to one week after session 4 was statistically significant (t10−t2 = 0.031, 95% CI 0.002-0.060, P = 0.027), whereas the other contrasts for stride length were not. FFD improved significantly in the short term, with significant immediate effects after sessions 1 and 2 (t3−t2 = 4.70, 95% CI 0.82-8.58, P = 0.005; t5−t4 = 4.40, 95% CI 0.53-8.28, P = 0.009), a significant average immediate effect across all four sessions (average immediate effect = 3.58, 95% CI 1.64-3.58, P < 0.001), and a significant positive linear trend across pre-session baselines. Improvements in FFD relative to the first pre-session were maintained immediately after session 4 and at one week, although the effect at one month was smaller and no longer statistically significant. Sensitivity analyses for secondary outcomes using AR(1) and CR2 produced directionally similar estimates to the primary models, but some inferences were sensitive to modeling assumptions in this small sample. In particular, the significance of selected acute and maintenance contrasts differed across AR(1) and CR2 for gait speed, stride length, and FFD, whereas overall patterns for SS5 were broadly consistent.

In an exploratory analysis, care level was not significantly associated with changes in TUG from the first pre-session assessment to post-session 4, one-week follow-up, or one-month follow-up (Spearman’s correlation coefficients were 0.131, 0.386, and 0.212, respectively; all not significant), suggesting no clear dose-response relationship by care level in this small cohort.

## Discussion

Novelty and significance of the present study

In the present study, the lumbar-type HAL-based therapy was associated with improvements in several aspects of physical function. TUG, gait speed, SS5, and FFD all showed short-term improvements from immediately before to immediately after session 1, and significant average within-session effects across the four sessions (Table 4). Moreover, for TUG, gait speed, and SS5, these improvements were maintained at both one week and one month after the fourth session.

Notably, each therapy session was limited to within 10 minutes (including donning and doffing the device), and the program consisted of just four sessions delivered once weekly, yet meaningful functional gains were still observed. In the context of a super-aged society, lumbar-type HAL-based therapy may therefore represent a promising strategy to support mobility and contribute to the extension of healthy life expectancy in older adults.

Importance of improving TUG time

In the present study, TUG time improved by approximately three to four seconds immediately after the four-session program, and this effect was maintained at both one week and one month after the final session (Table 4). The TUG captures several components of functional mobility, including sit-to-stand transfer, turning ability, and dynamic balance, and is widely used as a global index of mobility and fall risk in older adults [19]. TUG times ≥13.5 s have been associated with an increased risk of falls [20]; therefore, we included participants with TUG times ≥13.5 s in this study. Some studies have reported that falls decreased in parallel with improvements in TUG time [21]. Consequently, the observed improvement in TUG performance suggests that lumbar-type HAL-based therapy may enhance not only straight-ahead walking speed but also overall functional mobility and balance, with potential benefits for fall-risk reduction and independence in daily activities.

Functional domains related to the secondary outcomes

Gait speed is a simple, reliable, and responsive performance measure that integrates multisystem function. In the present study, improvements in gait speed relative to t2 were +0.166 m/s at t9, +0.170 m/s at t10, and +0.164 m/s at t11, all of which exceeded the 0.10 m/s threshold for a substantial change (Table 4). With respect to gait speed, a change of 0.05 m/s is regarded as a small but meaningful improvement, whereas a change of 0.10 m/s is considered a substantial change [22]. Therefore, our findings suggest that the substantial gains in gait speed achieved through the lumbar-type HAL-based therapy were maintained for at least one month after the final session. Gait speed has been termed a “functional vital sign,” and higher speed consistently reflects better global health and function [23]. In a pooled analysis of nine cohorts, gait speed independently predicted survival, with large gradients in 10-year mortality across the speed spectrum, underscoring its prognostic salience for patient-centered outcomes [24]. Gait speed is incorporated into diagnostic criteria for frailty, where slow gait is considered one of the central defining components of the frailty phenotype [25]. Because frailty is regarded as a potentially reversible condition, timely management and rehabilitation may enable individuals to transition back to a more robust state. In this context, implementing lumbar-type HAL-based therapy in frail older adults with reduced gait speed may help restore gait function and potentially facilitate a transition from frail to robust status, suggesting that lumbar-type HAL could also be a useful tool for frailty prevention and management.

In the present study, mean SS5 time also improved by approximately four to five seconds immediately after the four-session program, and this effect was maintained at both one week and one month after the final session (Table 4). The SS5 is widely used as a simple and reliable index of functional lower-limb muscle strength, transitional movements (sit-to-stand and stand-to-sit), balance control, and overall functional mobility in older adults [26]. Poor performance on sit-to-stand tests has been associated with impaired lower-extremity function, future disability, and an increased risk of recurrent falls, and the SS5 has been incorporated into clinical tools for fall-risk stratification in community-dwelling older adults [27]. Because the HAL-based therapy protocol in this study consisted primarily of repetitive sit-to-stand movements performed while wearing the lumbar-type HAL, the training task closely matched the movement pattern required in the SS5. Thus, lumbar-type HAL-based therapy may provide particular benefits for improving functional mobility during transfers and potentially reducing fall risk in older adults with reduced physical function.

In contrast, FFD showed a significant improvement immediately after completion of the four-session program, but this effect was no longer statistically significant at the one-month follow-up (Table 4), indicating that the gain in forward flexion flexibility was transient. FFD is a simple clinical test of maximal forward trunk bending that reflects global spinopelvic mobility and hamstring flexibility and has shown good validity, reliability, and responsiveness [18]. Given that the present HAL-based therapy included repeated trunk flexion and extension and squat-like movements performed with interactive feedback at the lumbopelvic region, it is plausible that this HAL-based therapy temporarily enhanced spinopelvic mobility and hamstring extensibility, thereby improving FFD [28]. In this context, lumbar-type HAL may offer the practical benefit of enabling older adults with reduced physical function to practice forward-bending activities, such as reaching to the floor and managing footwear. However, because the effect on FFD was not maintained at one month, longer or more frequent HAL-based therapy protocols and extended follow-up will be necessary to determine whether flexibility-related benefits can be sustained over time.

Characteristics of lumbar-type HAL compared with conventional rehabilitation

In the present study, a very low-dose lumbar-type HAL training protocol (approximately 10 minutes per session, once weekly for four weeks; total HAL-based therapy time, approximately 40 min) produced clinically meaningful improvements in gait speed, TUG, SS5, and FFD (Table 4). Notably, no participants withdrew after the initiation of therapy. In a previous short-term (five-week) HAL study with a mean participant age of approximately 75 years [12], only one of 40 participants in the HAL group dropped out. In addition, a study in frail patients who performed exercise with HAL also suggested the safety of HAL-based training [29]. Taken together, these findings suggest that even older adults, who are often frail, can tolerate short-duration HAL-based therapy. Conventional multicomponent exercise programs for frail or sarcopenic older adults typically prescribe two to five supervised sessions per week over 12-24 weeks, with 45-70 min per session, to achieve clinically meaningful gains in physical performance and reversal of frailty [30]. Furthermore, dose-response trials suggest that higher training volumes and intensities generally yield larger improvements in lower-limb strength and walking endurance in frail older adults [31]. Direct comparisons with such programs cannot be made from our single-arm study. We therefore view our findings as preliminary and hypothesis-generating: lumbar-type HAL may potentially support task-specific practice (e.g., sit-to-stand, squatting, trunk movements) in some mobility-limited older adults, which could contribute to short-term functional improvements. This interpretation is consistent with previous studies in patients with chronic heart failure and mobility-limited older adults, in which lumbar HAL-based therapy produced superior gains in knee extensor strength and mobility outcomes over relatively short periods [12,32]. Interactive biofeedback therapy is thought to induce rapid changes in movement patterns, which may explain the immediate functional improvements observed after short-duration HAL-based therapy [10]. Short-term neuromotor priming or transient biomechanical facilitation gained by HAL-based training may contribute to the immediate pre-post improvements [33]. Accordingly, the immediate effects observed in the present study may reflect priming and task familiarization. The more durable component might be better reflected at later time points (e.g., t9, t10, and t11). Future controlled trials will be needed to determine whether lumbar-type HAL improves rehabilitation efficiency and to clarify the underlying mechanisms. Such studies may also help elucidate how the immediate effects of HAL relate to longer-term improvements in physical function.

Because HAL is costly and currently available at only a limited number of medical institutions, access for the general population remains restricted. Expanding availability and improving affordability will be important future challenges to enable broader clinical use.

Limitations

This study has several limitations. First, it was a small, single-center, single-arm intervention study (n = 10) without a control group, which substantially limits causal inference. The observed improvements may reflect learning or practice effects from repeated testing, regression to the mean, or concurrent rehabilitation activities provided as part of routine day-care services, rather than the lumbar-type HAL therapy itself. Accordingly, our findings should be interpreted as preliminary and hypothesis-generating. Second, the wide age range (65-96 years) likely introduced heterogeneity in baseline function and responsiveness to training, which may have contributed to variability in outcomes and limits generalizability. Furthermore, disease heterogeneity may also have influenced responsiveness to HAL-based therapy. Participants had diverse underlying conditions (e.g., post-stroke sequelae, orthopedic disorders, and chronic medical diseases), which can impose different primary constraints on mobility. Additionally, the generalizability of this study is limited by the fact that the participants were restricted to Japanese individuals and that information on body mass index (BMI) was not available. Third, outcome assessors were not blinded. Given the repeated testing and short intervention duration, performance and measurement bias cannot be excluded. These sources of heterogeneity and potential bias may have increased inter-individual variability and contributed to differential improvements across outcomes. Finally, although some of the beneficial effects were maintained up to one month after the final session, longer-term outcomes beyond this period remain unknown. Further studies are required to determine whether the effects of lumbar HAL on mobility and physical function can be sustained over longer follow-up periods and to clarify the potential benefits of continuing HAL-based therapy for several months.

## Conclusions

In the present study, a short-duration interactive biofeedback therapy delivered with a lumbar-type HAL was associated with improvements in TUG time, gait speed, and SS5 time, and these changes were maintained for up to one month after the final session. Given the limited sample size and the absence of a control group, these findings should be interpreted cautiously and considered hypothesis-generating. Nevertheless, lumbar-type HAL-based therapy may be a feasible, low-burden approach for older adults with reduced physical capacity. Future research should evaluate the effects of HAL in randomized controlled trials with a larger sample size and longer follow-up.
